# Cancer insights from magnetic resonance spectroscopy of cells and excised tumors

**DOI:** 10.1002/nbm.4724

**Published:** 2022-03-25

**Authors:** Marie-France Penet, Raj Kumar Sharma, Santosh Bharti, Noriko Mori, Dmitri Artemov, Zaver M. Bhujwalla

**Affiliations:** 1 Division of Cancer Imaging Research, The Russell H. Morgan Department of Radiology and Radiological Science, Baltimore, Maryland, USA; 2 Sidney Kimmel Comprehensive Cancer Center, Baltimore, Maryland, USA; 3 Department of Radiation Oncology and Molecular Radiation Sciences, The Johns Hopkins University School of Medicine, Baltimore, Maryland, USA

**Keywords:** cancer, metabolism, multinuclear MRS, stromal cells

## Abstract

Multinuclear ex vivo magnetic resonance spectroscopy (MRS) of cancer cells, xenografts, human cancer tissue, and biofluids is a rapidly expanding field that is providing unique insights into cancer. Starting from the 1970s, the field has continued to evolve as a stand-alone technology or as a complement to in vivo MRS to characterize the metabolome of cancer cells, cancer-associated stromal cells, immune cells, tumors, biofluids and, more recently, changes in the metabolome of organs induced by cancers. Here, we review some of the insights into cancer obtained with ex vivo MRS and provide a perspective of future directions. Ex vivo MRS of cells and tumors provides opportunities to understand the role of metabolism in cancer immune surveillance and immunotherapy. With advances in computational capabilities, the integration of artificial intelligence to identify differences in multinuclear spectral patterns, especially in easily accessible biofluids, is providing exciting advances in detection and monitoring response to treatment. Metabolotheranostics to target cancers and to normalize metabolic changes in organs induced by cancers to prevent cancer-induced morbidity are other areas of future development.

## 1 | INTRODUCTION

Applications of magnetic resonance spectroscopy (MRS) to investigate cancer can be traced to studies in the 1970s that used ^1^H MRS to characterize water relaxation of neoplastic cells and tissues at field strengths of 30 MHz.^1,2^ These early studies were followed by ^31^P MRS characterization of intact cells^3^ and tumor tissues^4^ performed at higher field strengths that identified the metabolic-rich spectra of cancer cells and tumor extracts.^5–7^ The feasibility of measuring pH from the chemical shift of inorganic phosphate^8^ was another important advance in understanding tumor pH. Breakthrough studies demonstrating the feasibility of in vivo ^31^P MRS of cancers significantly expanded the field,^9,10^ stimulating applications of multinuclear MRS in understanding cancer physiology and metabolism, and in identifying response to cancer therapy.^11,12^ The 1990s also saw the application of pattern recognition and the use of neural networks, forerunners of current artificial intelligence (AI) approaches, to analyze high-resolution spectra obtained from tissues and biofluids to assist in the detection and classification of cancers.^13,14^

Based on the foundations laid by these and other studies, MRS of cancer has evolved into a field with multiple specialized focus areas such as multinuclear in vivo spectroscopy and spectroscopic imaging of tumors, spectroscopy of intact perfused cells, and ex vivo high-resolution spectroscopy of cells and tissue extracts, all of which have impacted cancer discovery and treatment. Advances in novel sequence design and spectral editing,^15–17^ coil designs,^18^ higher field strengths,^19^ and novel contrast such as chemical exchange saturation transfer (CEST),^20,21^ dynamic nuclear polarization of ^13^C,^21–23^ and the availability of hybrid PET-MR technology^24^ have transformed the field over the past decade, and will continue to do so well into the future.

**FIGURE 1:**
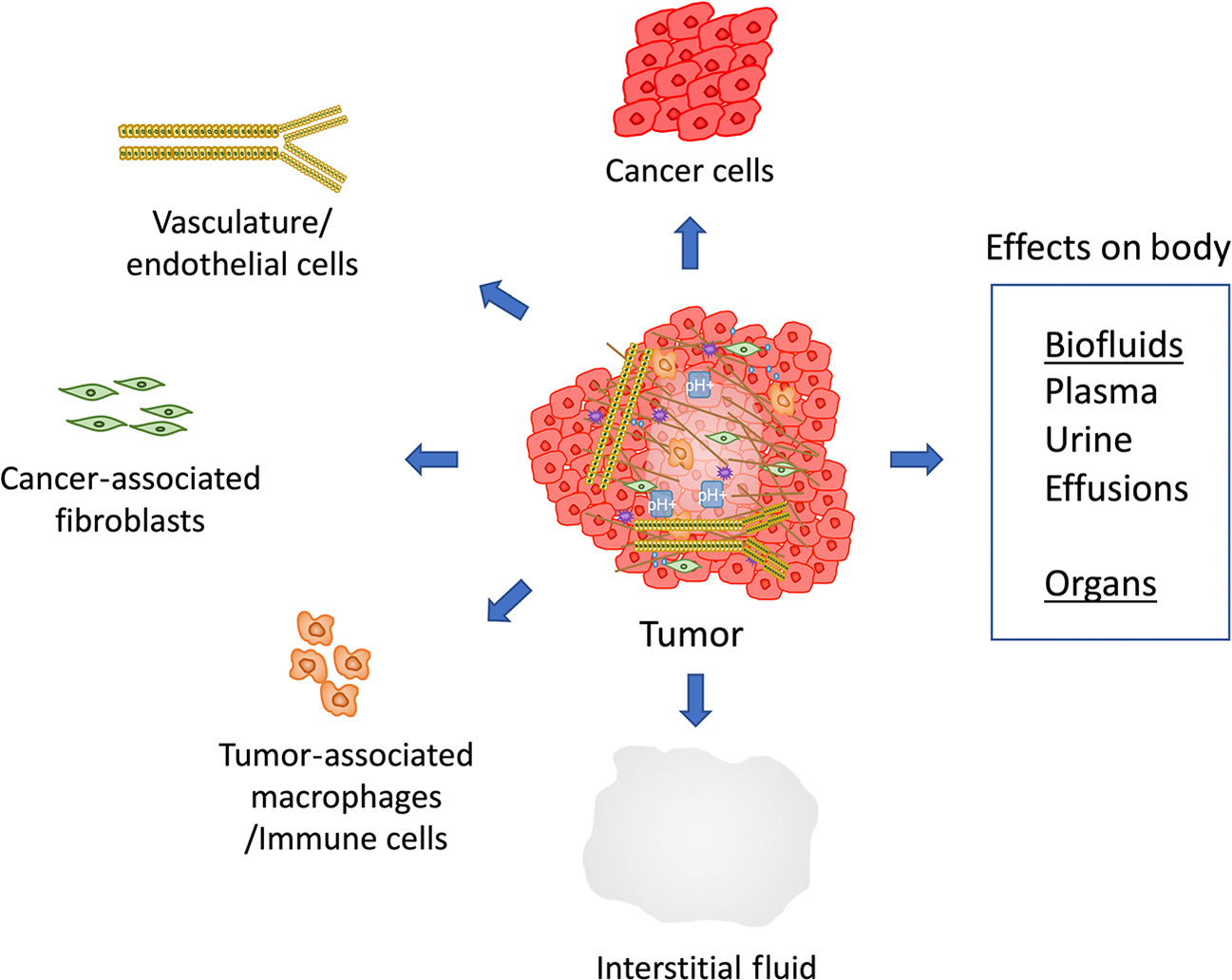
Schematic of some of the different components of the tumor microenvironment. A solid tumor consists of cancer cells as well as an array of stromal cells such as cancer-associated fibroblasts, tumor-associated macrophages, vascular and lymphatic endothelial cells, and various immune cells. Graphic designed by Dr. Samata Kakkad

The purpose of this review is to outline the impact of MRS of extracted cells, tissues, and biofluids, in the discovery, detection, and treatment of cancer, and to provide a perspective on exciting new directions for the future. The metabolome has been the primary domain of ex vivo MRS applications. With its minimal sample preparation requirements, reproducibility, and quantitative ability, ex vivo MRS is a technique of choice for investigating the metabolome of cells, tissues, and biofluids in cancer. As outlined in the schematic in Figure 1, a tumor consists of cancer cells as well as an array of stromal cells such as cancer-associated fibroblasts (CAFs), tumor-associated macrophages (TAMs), vascular and lymphatic endothelial cells, and various immune cells. The cancer secretome, contained in the tumor interstitial fluid, and tumor vasculature, form additional components of the tumor ecosystem. Because of the complexity of the tumor microenvironment that is characterized by abnormal vasculature, hypoxia, and acidic extracellular pH (pHe), with some or all of these characteristics occurring together, ex vivo MRS has also provided unique opportunities to understand the impact of these abnormal physiological environments under carefully controlled conditions. Ex vivo MRS studies of genetically engineered cell lines with specific pathways silenced or overexpressed have also highlighted the impact of these pathways on the metabolome. Cancers also significantly impact the metabolism of other organs and these changes also contribute to metabolic changes in biofluids such as plasma, urine, and effusions that can be investigated with ex vivo MRS. Here, we have summarized insights gained from ex vivo MR spectroscopy of the tumor metabolome and of different tumor compartments.

## 2 | CANCERS AND CANCER CELLS

### 2.1 | Human tumor xenografts and human cancers

Metabolic characterization with MRS of human tumor xenografts and human cancers has been performed with cells derived from several cancer types including brain, breast, pancreatic, prostate, ovarian, renal, liver, and colon cancers,^25–31^ and with excised human tissue.^30,32^ These studies have identified dysregulated choline, glutamine, and glucose metabolism as metabolic hallmarks of cancer.^25–27,33,34^ Additionally, loss of wild-type isocitrate dehydrogenase (IDH) and the resultant production of 2-hydroxyglutarate (2-HG) identified in high-resolution magic angle spinning (HR MAS) studies of low-grade glioma^35^ have resulted in in vivo measurements of 2-HG to identify gliomas with a more favorable prognosis.^36^

Altered choline metabolism in cancer, identified by MRS, was reported in early studies with human colon and breast cancer xenografts,^5^ Friend leukemia and a mouse fibrosarcoma,^37^ and glioma.^38^ Our interest in choline metabolism began with observations that human breast cancer cells transfected to express a metastasis suppressor gene, nm23, displayed significant changes in choline metabolism compared with empty vector transfected cells and tumors.^39^ We subsequently characterized choline metabolites in a panel of normal senescent, immortalized, and malignant breast cancer cells to further confirm aberrant choline metabolism in breast cancer, and demonstrated that malignant transformation, but not cellular proliferation, altered choline metabolism.^40^ Since then, we have confirmed dysregulated choline metabolism in a panel of prostate cancer cell lines,^27^ and a panel of pancreatic cancer cell lines and tumors.^26^ A similar characterization has been performed with a panel of ovarian cancer cells^25^ and brain cancer cells.^28^ The most commonly observed changes in choline metabolism are increased phosphocholine (PC) identified in high-resolution extract spectra that leads to an increase of total choline (tCho) in vivo. This increased tCho signal in vivo has led to the development of ^1^H MRS of tCho being used to detect cancers in humans.^41^ Ex vivo MRS studies also identified the role of the tumor microenvironment in modulating choline and lipid metabolism.^42^ The causes of altered choline metabolism in cancer are primarily increased expression of choline kinase (Chk),^43,44^ the enzyme that converts choline to PC, and increased expression of choline transporters.^45^ Activation of phosphatidylcholine-specific phospholipase C, an enzyme implicated in cell signaling, cell cycle regulation, and cell proliferation, has also been shown to play a role in the increase of intracellular PC detected in some cancer cells, such as ovarian cancer cells.^46^ Chk inhibitors are being developed for cancer treatment,^47,48^ including competitive Chk inhibitors at the choline-binding site.^49^

We found that downregulation of Chk using small interfering RNA (siRNA-Chk) significantly reduced proliferation in breast cancer cells^50,51^ and tumors.^52^ Combining siRNA-Chk with 5-FU treatment resulted in additional reduction of cell viability and proliferation in breast cancer cells.^51^ Recently, a novel potent and selective small-molecule Chk-α inhibitor, V-11–0711, which acts by inhibiting the catalytic activity of Chk-α,^53^ was evaluated in HeLa cells. V-11–0711 significantly reduced PC, as detected by liquid chromatography–tandem mass spectrometry, but did not cause cell death, unlike downregulation of Chk by siRNA-Chk that caused apoptosis.^53^ We treated triple negative breast cancer cell lines with V-11–0711 that significantly reduced PC levels but did not reduce cell proliferation.^54^ Reduction of PC had little effect on the proliferation of breast cancer cells as long as Chk protein levels were not reduced, indicating the importance of the noncatalytic role of Chk in cancer through the formation of a protein complex with epidermal growth factor receptor (EGFR) and c-Src.^55^ These results support the development of treatment strategies that destabilize or downregulate Chk in breast cancer.

**FIGURE 2:**
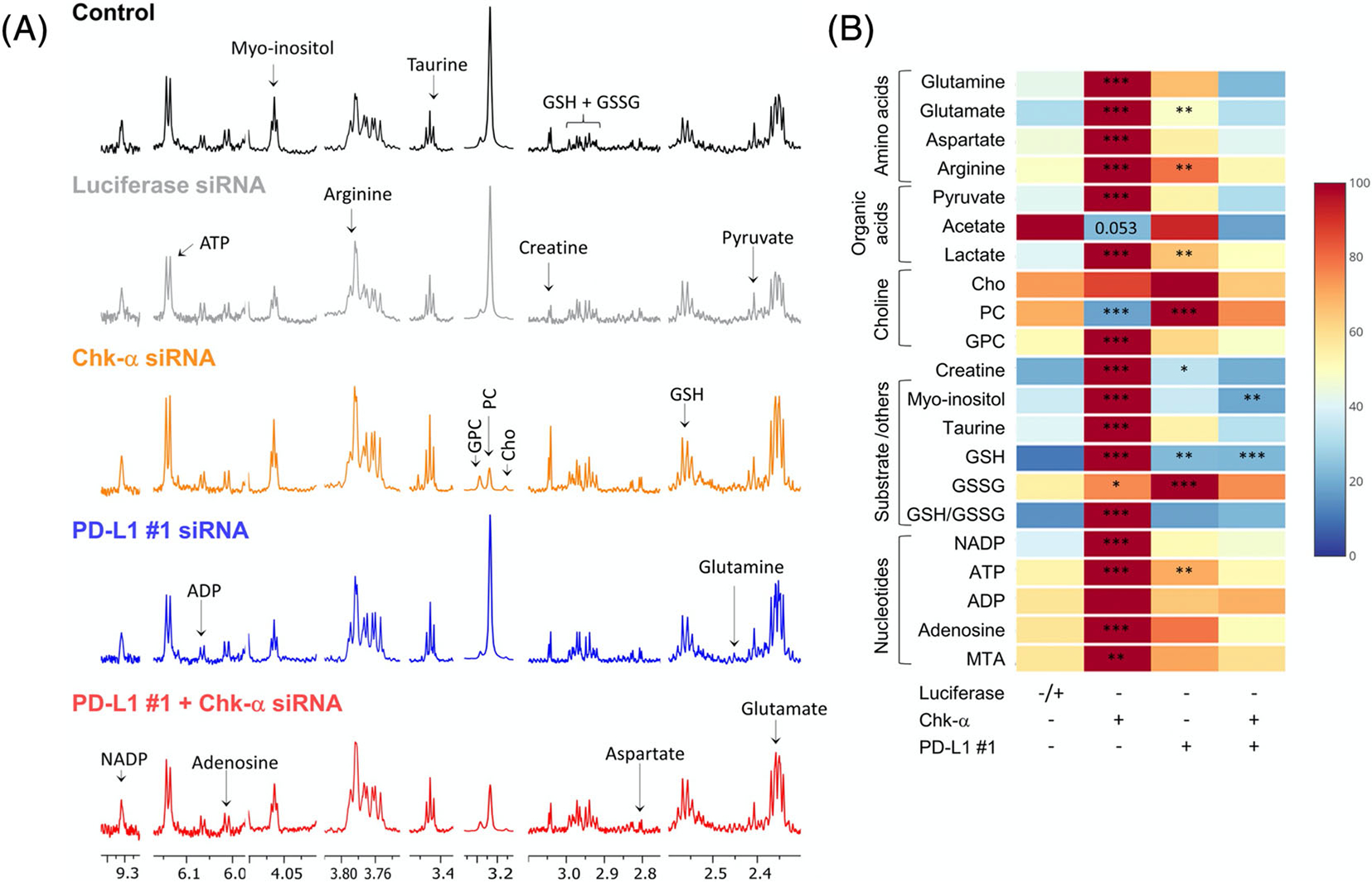
Metabolic effects of program death ligand 1 (PD-L1) and choline kinase (Chk) downregulation as detected by ^1^H MRS. (A) Representative high-resolution ^1^H MR spectra obtained from the aqueous phase of triple negative breast cancer MDA-MB 231 cells; untreated (black), treated with luciferase small interfering RNA (siRNA) (gray), treated with Chk-α siRNA (orange), treated with PD-L1 siRNA (blue), and treated with PD-L1 and Chk-α siRNA (red). GSH, glutathione; GSSG, oxidized glutathione; MTA, S-methyl-5′-thioadenosine. (B) Metabolic heat map generated from quantitative analysis of ^1^H MR spectral data of the aqueous phase, displaying differences in the metabolic profile of MDA-MB-231 cells with the different siRNA treatments. *p ≤ 0.05, **p ≤ 0.01, ***p ≤ 0.001, compared with the control group. Reproduced in accordance with the Creative Commons CC BY license from^57^

Cancer cells have been shown to evade the host immune system by expressing high levels of immune inhibitory signaling proteins, such as programmed death ligand 1 (PD-L1).^56^ In recent studies, downregulation of the immune checkpoint PD-L1 was found to increase Chk in human breast and pancreatic cancer cells, and downregulation of Chk was found to increase PD-L1. This interaction was eliminated in cells with Chk or cyclooxygenase (COX-2) silenced, suggesting that choline metabolism and inflammation played a role in this inverse relationship.^57^ Downregulation of PD-L1 resulted in significant changes in several metabolites detected by ^1^H MRS, identifying for the first time previously unknown roles of PD-L1 in cancer cell metabolic reprogramming (Figure 2).^57^

Similar to PC, elevated phosphoethanolamine (PE) is also frequently observed in MRS studies of human cancers and xenografts.^58^ Because culture medium does not usually contain ethanolamine, PE levels in spectra obtained from cells in culture are always low. The molecular causes underlying the increase of PE in cancers and the role of PE in cell survival are relatively underexplored. In studies investigating the roles of ethanolamine kinases (Etnk-1 and −2), we found that breast and pancreatic cancer cells showed higher PE compared with their nonmalignant counterparts, with Etnk-1 a major cause of the elevated PE levels in these cancer cells. There was little or no contribution from Chk-α, Chk-β, or Etnk-2.^59^ Downregulation of Etnk-1 with siRNA significantly reduced cell viability, suggesting that Etnk-1 may be a potential therapeutic target in breast and pancreatic cancers.^59^

Cancer cells in culture proliferate with aggressive phenotypic characteristics despite the depletion of ethanolamine in the medium suggesting that, like PC, a decrease of PE does not appear to be critical for cancer cell viability. Like Chk, Etnk-1 may have a noncatalytic role in the scaffolding of protein complexes, competitive protein interactions, allosteric effects on other enzymes, subcellular targeting, and DNA binding.^60^ Higher expression of Etnk-1 in cancer cells may lead to the development of an ethanolamine radiotracer analogous to ^11^C-choline to detect cancer.^61^

Glutamine, a conditionally essential amino acid, has long been observed to play an important role in cancer metabolism.^62^ It is a precursor for the biosynthesis of proteins, nucleotides, and amino sugars, plays a role in the scavenging of NH_3_ in the urea cycle, and is used for energy production in the tricarboxylic acid (TCA) cycle.^63^ “Glutamine addiction” of cancer cells was observed as early as 1955.^64^ Pancreatic ductal adenocarcinoma (PDAC) cells have been identified to be glutamine avid that was attributed to an increase of the SLC1A5 glutamine transporter.^65^ Mutant *Kras* that occurs in almost 90% of PDAC is a major cause of glutamine addiction.^66^ Cancer cells rapidly convert glutamine to glutamate because of the high expression of mitochondrial glutaminase (GLS). Glutamate is metabolized to α-ketoglutarate through glutamate dehydrogenase and enters the TCA cycle for the production of pyruvate and ATP.

SLC1A5, also known as ASCT2 (for alanine, serine, cysteine transporter 2), is a neutral amino acid transporter that is localized in the plasma membrane.^67,68^ Glutamine is the preferred substrate for SLC1A5.^68,69^ SLC1A5 is being actively investigated as a pharmacological target in cancer.^68^ The protein is expressed in lung, skeletal muscle, large intestine, kidney, testis, T-cells, brain, and adipose tissue, but it is significantly upregulated in cancers.^69^ GLS is an amidohydrolase enzyme that generates glutamate from glutamine.^63^ There are four known isoforms of GLS. GLS1 encodes two types of kidney-type GLS with a high activity and low Michaelis constant Km. GLS2 encodes two forms of liver-type GLS with a low activity and allosteric regulation.^70^ Pharmacological inhibition of GLS was found to be effective in a subset of PDAC.^71^ GLS inhibition of pancreatic cancer cells with CB-839 resulted in significant antiproliferative activity in cells, but compensatory metabolic networks reduced antitumor effects in an in vivo tumor model.^72^ In a recent ^13^C MRS study, glutamine utilization in tumors was studied following infusion with ^13^C-enriched glutamine.^34^ Two patient-derived xenograft (PDX) models of breast cancer were used, luminal-like/ER + MAS98.06 and basal-like/triple-negative MAS98.12. Inhibition of tumor growth after treatment with CB-289 was observed in MAS98.06 tumors, but not in MAS98.12 tumors. To assess the metabolic effects of CB-839, uptake and conversion of glutamine were determined using HR MAS MRS of excised tumor tissues after intravenous infusion of [5-^13^C] glutamine following 2 days of CB-289 treatment. The study showed an accumulation of glutamine in both tumor models post-treatment. CB-839 also caused depletion of alanine, proline, and glutamate in the MAS98.06 model.^34^

In vivo CEST-based techniques have been developed to detect glutamate, glucose, glycogen, creatine, myo-inositol, glycosaminoglycans, and mobile proteins and peptides rich in amide protons.^73^ Compared with ^1^H MRS, glutamate-weighted CEST MR imaging (GluCEST MRI) offers a more sensitive detection mechanism free of glutamine interference.^74^

Ex vivo MRS of cells engineered to overexpress or downregulate genes, and tumors derived from these cells, have been valuable in understanding the role of these genes and pathways in altering metabolism. COX-2 downregulation in poorly differentiated MDA-MB-231 human breast cancer cells using short hairpin siRNA expression resulted in a significant decrease in PC and tCho that was detected by ^1^H MRS. A significant increase in lipids was also observed.^75^ Loss of p53 in colon cancer cells resulted in an increase in PC and tCho.^76 1^H MRS analysis of noninvasive MCF-7 breast cancer cells overexpressing Chk showed significantly higher PC and elevated triglyceride signals associated with a more invasive phenotype.^77^

Mutations in IDH1 have been reported in more than 70% of low-grade gliomas and secondary glioblastomas. ^1^H MRS was applied to investigate wild-type IDH1 and mutant IDH1 glioma cells. Reduction of glutamate, lactate, and PC and elevation of 2-HG were observed in mutant IDH1 cells compared with wild-type cells.^78^ Hyperpolarized ^13^C MRS applied to monitor pyruvate dehydrogenase activity identified a reduction in the metabolism of hyperpolarized 2-^13^C pyruvate to 5-^13^C glutamate, and a reduction in the glucose flux to glutamate in IDH1 mutant cells.^79^

**FIGURE 3:**
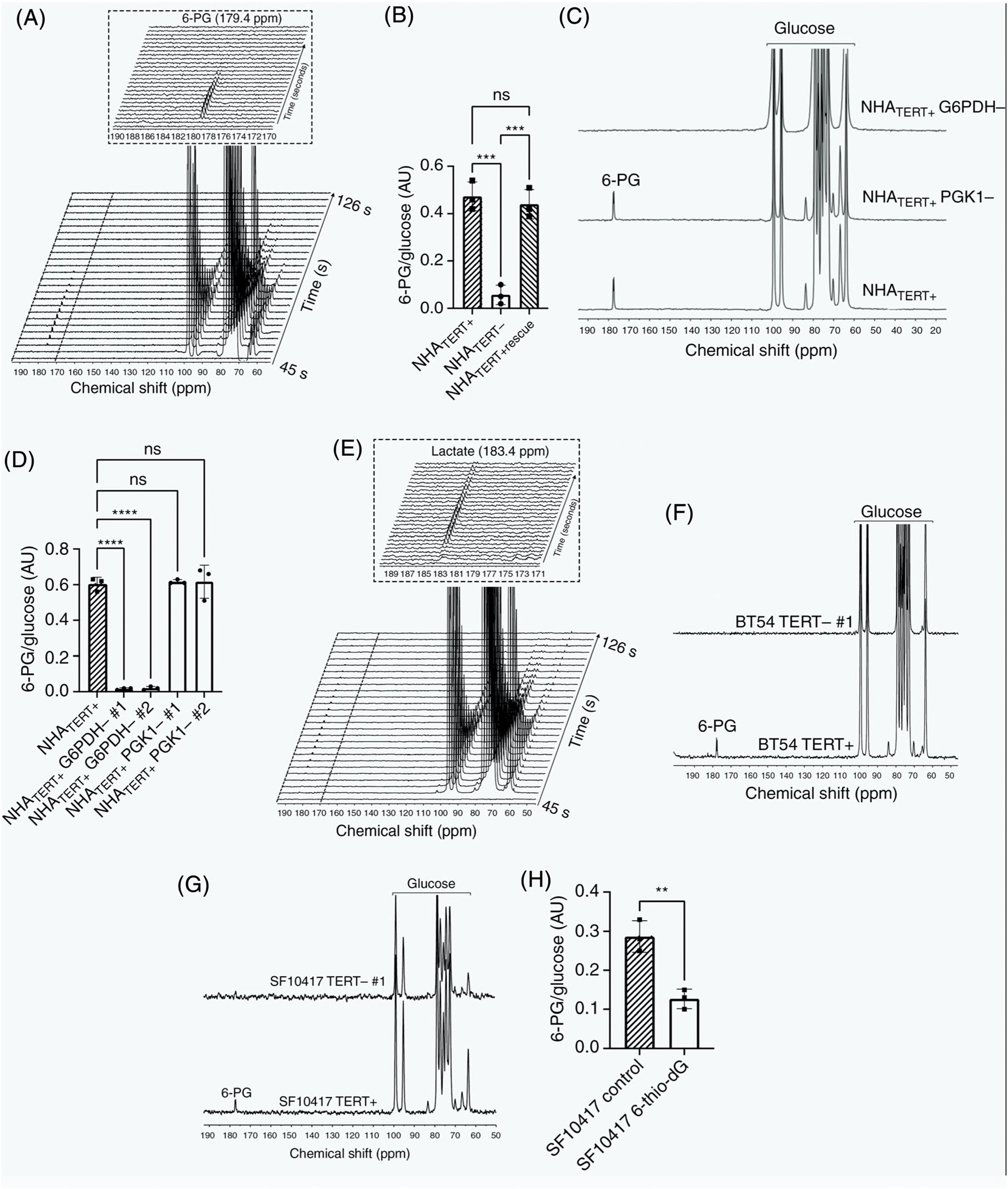
Telomerase reverse transcriptase (TERT)-linked increase in pentose phosphate pathway (PPP) flux noninvasively monitored using hyperpolarized [U-^13^C, U-^2^H]-glucose in low-grade oligodendrogliomas (LGOG) cells. (A) Representative spectral array of hyperpolarized [U-^13^C, U-^2^H]-glucose metabolism to 6-phosphogluconate (6-PG) in normal human astrocytes (NHA)_TERT+_ cells. (B) 6-PG/glucose ratio in the doxycycline-inducible NHA_TERT+_ model. Representative summed ^13^C-MR spectra (C) and quantification of 6-PG production from hyperpolarized [U-^13^C, U-^2^H]-glucose (D) in NHA_TERT+_ cells in which glucose-6-phosphate dehydrogenase (G6PDH) or phosphoglycerate kinase 1 (PGK1) has been silenced. (E) Representative spectral array of hyperpolarized [U-^13^C, U-^2^H]-glucose metabolism to lactate in NHAs that lack TERT and use the ALT pathway (NHA_ALT_) cells. Representative summed ^13^C spectra showing the effect of TERT silencing on hyperpolarized [U-^13^C, U-^2^H]-glucose metabolism in the BT54 (F) or SF10417 (G) models, both derived from LGOG patients. (H) Effect of 6-thio-2′-deoxyguanosine (6-thio-dG) on the 6-PG/glucose ratio in SF10417 cells. ^**^p < .01, ^***^p < .005, ^****^p < .0001, ns = non-significant. Reproduced with permission from^80^

Telomerase reverse transcriptase (TERT) has been shown to be essential for tumor proliferation, including in low-grade oligodendrogliomas (LGOGs). Immortalized normal human astrocytes with doxycycline-inducible TERT silencing were studied to understand the relationship between TERT and glucose metabolism using 2-^13^C glucose MRS of cell extracts and hyperpolarized U-^13^C, U-^2^H-glucose MRS of live cells.^80^ Patient-derived LGOG cells, orthotopic tumors, and LGOG patient biopsies were also analyzed. The study showed that TERT upregulates glucose flux and increases antioxidant capacity (Figure 3). Hyperpolarized [U-^13^C, U-^2^H]-glucose flux can be used for noninvasive imaging of TERT expression in preclinical LGOG models in vivo.^81^

### 2.2 | ^1^H MR MAS of human cancers

An important advance in ex vivo MRS of cancer, especially human cancers, occurred with the application of magic angle spinning to ex vivo tumor tissue.^82^ HR MAS MR spectroscopy enables analysis of intact tissue with minimal sample preparation, allowing the combination of metabolite measurements with genomic, proteomic, and histologic data. Studies have been performed on different tumor types, including brain,^35,83,84^ breast,^85^ prostate,^86,87^ cervix,^88^ and pancreas.^89^ HR MAS has also been applied to analyze transgenic tumor models such as a medulloblastoma that originated from a transgenic mouse overexpressing the smoothened (SMO) receptor in granule cell precursors,^90^ and a pancreatic tumor model with a Kras mutation.^89^

^1^H HR MAS applied to pancreatic tissue samples from mouse models and patients consistently identified decreased lipids in pancreatitis and in biopsies from invasive cancer compared with normal tissue. Lactate and taurine levels remained unchanged in inflammation but increased in tumor samples.^89^ The technique was also used to measure metabolites in breast cancer tissue. Tumor samples were identified from noninvolved samples with a sensitivity of 82%, and a specificity of 100% based on relative intensities of glycerophosphocholine (GPC), PC, and choline signals.^85^ Choline-containing metabolites levels increased from preinvasive to invasive cervical cancer in a study that combined ^1^H and ^31^P HR MAS.^88^ The study compared histologically normal cervix, cervical intraepithelial neoplasia, either mild or moderate/severe, and invasive cancer. PE increased in cancer compared with normal tissue. The comparison of normal tissue from cancer patients with normal tissue from noncancer patients showed a reduction of alanine and creatine in normal tissue from cancer patients.^88^

HR MAS has been applied for prognosis, as shown in locally advanced rectal cancer,^91^ to predict response to therapy,^92^ and to assess response to treatment, as shown in prostate cancer, to follow the effects of gonadotrophin-releasing hormone blocker.^93^

The ability to analyze an intact biopsy specimen allows investigation of the reactive stromal compartment, when present, by histology.^94^ Reactive stromal content can be defined from the percentage of reactive stroma identified by trichrome staining.^95^ Prostate cancer reactive stroma is characterized by disorganized and much smaller fibers composed of a myofibroblast/fibroblast mix with a significant decrease or complete loss of fully differentiated smooth muscle accompanied by an elevation in expression of collagen type I, tenascin, and fibroblast activation protein,^95^ whereas normal prostate stroma is predominantly smooth muscle. High reactive stromal content was associated with biochemical recurrence. In a study where prostate cancer samples were divided based on low and high reactive stromal content, the analysis of 23 metabolites showed significant differences between low and high reactive stromal content in the levels of spermine, citrate, taurine, leucine, and scyllo-inositol. Lower levels of citrate and spermine and higher levels of leucine, taurine, and scyllo-inositol were detected in high reactive stromal content samples.^94^ The metabolic profile observed appeared to be linked to inflammation and extracellular matrix (ECM) remodeling.

**FIGURE 4:**
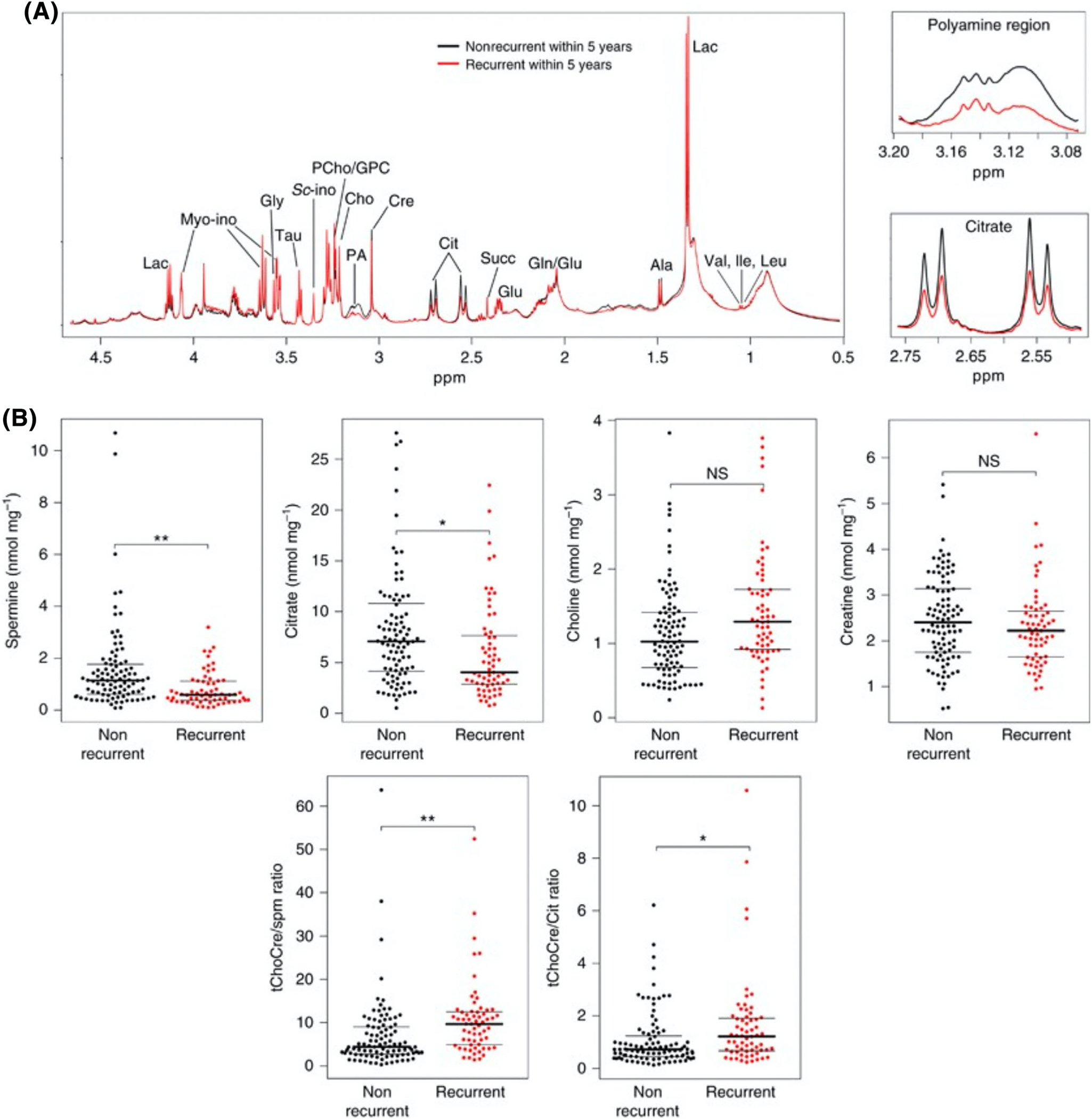
Ex vivo metabolic analysis of biomarkers predictive of prostate cancer recurrence following radical prostatectomy. (A) Average high-resolution magic angle spinning (HR MAS) MR spectra of tumors from nonrecurrent (black) and recurrent (red) prostate cancer groups. Magnifications of the polyamine (containing spermine and putrescine) and citrate regions are shown. (B) Quantified peak integrals of spermine, citrate, choline, and creatine (nmol/mg), and tChoCre/Spm and tChoCre/Cit ratios are shown as beeswarm plots (*n* = 158). The thin black horizontal lines represent the 25% and 75% quartiles, and the thick black horizontal lines represent the median value (*p < 0.05, **Q < 0.05). Ala, alanine; Cho, choline; Cit, citrate; Cre, creatine; Gln, glutamine; Glu, glutamate; Gly, glycine; Lac, lactate; Myo-ino, myo-inositol; NS, nonsignificant; PA, polyamines; PC/GPC, phosphocholine/glycerophosphocholine peaks; *Sc*-ino, *scyllo*-inositol; Spm, spermine; Succ, succinate; Tau, taurine. Reproduced in accordance with the Creative Commons CC BY license from^96^

In a retrospective study using HR MAS MRS of tissue samples obtained from radical prostatectomy specimens, metabolic differences between recurrent and nonrecurrent tumors were investigated (Figure 4).^96^ High levels of spermine and citrate were associated with longer recurrence-free survival, while high tCho+creatine/spermine and higher tCho+creatine/citrate ratios were associated with shorter time to recurrence.^96^

### 2.3 | Multivariate data analysis of MR spectra

Because of the complexities of high-resolution ex vivo MRS data, automated multivariate analysis methods such as unsupervised principal component analysis (PCA) and supervised partial least discrimination analysis (PLS-DA) are applied to extract information from the spectroscopy data to identify key metabolic changes that occur in biochemical pathways.^97–99^ Multivariate analysis reduces data dimensionality so that data can be presented as clusters of samples from each group.^100^

PCA is a multivariate unsupervised regression method used to identify the maximum variance present in complex data^101^ such as high-resolution MR spectra of tissue and extracts and biofluids.^102,103^ Unlike univariate analysis that requires identifying and analyzing individual peaks, information on patterns and classification can be obtained without prior knowledge of group-specific information.

In PCA the algorithm converts data to lower dimensional subspace to provide a projection of data in two- or three-dimensional space.^104^ These projections are weighted outcomes of the principal components that decide the principal direction of variations.^97^ PC1 and PC2 represent the first and second principal directions with the largest variation in the data. Two- and three-dimensional visualization of data can identify outliers present in the data because of inhomogeneity of sample preparation, instrumental error, or other sample variability.

PLS-DA is a supervised metabolomics data analysis method that is similar to other supervised approaches such as neuronal network and random forest analysis.^105^ Like PCA, data are converted to a lower dimension space to optimize the separation among groups using class information and variable data. Unlike PCA, PLS-DA is prone to overfitting of data.^106^ Therefore, the accuracy of this method is measured by separating data into training and test datasets to validate the model. Different crossvalidation (CV) methods such as leave one out CV and Monte Carlo CV are used to demonstrate the predictive ability of the model.^97,105,107,108^

Loading plots, variable importance plots, and regression coefficients obtained from PLS-DA statistical analysis are important in the identification of altered variables. Several automated multivariate analysis software for metabolomics data exploration such as Simca (Umetrics Inc, Kinnelon, NJ), Unscrambler X (Camo Analytics, Norway), and Metaboanlyst^109^ are available. Multivariate statistical methods have been successfully applied in identifying biomarkers in diseases such as glioma^110^ and lung cancer.^111^

## 3 | STROMAL CELLS

### 3.1 | Cancer-associated fibroblasts

Ex vivo MRS of stromal cells can provide important insights into their metabolism and their response to physiological environments such as hypoxia and acidic pHe found in tumor microenvironments. CAFs play an important role in immune suppression, the formation of the ECM, and tumor progression.^112^

In human lung fibroblasts studied with ^1^H MRS to understand the impact of aging,^113^ cholesterol and lipid signals were found to increase from nonsenescent to senescent cells. In another study, ^13^C MRS was applied to investigate mitochondrial fuel metabolism in human fibroblasts in culture.^114^ Primary fibroblasts from five healthy subjects and six patients with mutated pyruvate dehydrogenase complex (PDC) were grown in media with or without dichloroacetate in the presence of ^13^C-labeled glucose, allowing measurement of the rates of glucose consumption and lactate production. The study showed that five of the six PDC-deficient cells had significantly higher glucose consumption, lactate production, and label-derived acetyl-CoA, indicative of increased glycolysis compared with the controls.^114^

To understand the influence of hypoxia that is frequently observed in prostate cancer in modifying fibroblast metabolism, ^1^H MRS was used to investigate hypoxia-mediated metabolic changes in human prostate fibroblasts (WPMY-1) and in human prostate cancer-associated fibroblasts (PCAFs).^115^ Significant differences in the metabolic profiles were identified between WPMY-1 and PCAFs under normoxic conditions. The metabolic response to hypoxia was very different for both cell lines, with increased lipid signals in PCAFs, and increased water-soluble metabolites in WPMY-1.

### 3.2 | Macrophages

**FIGURE 5:**
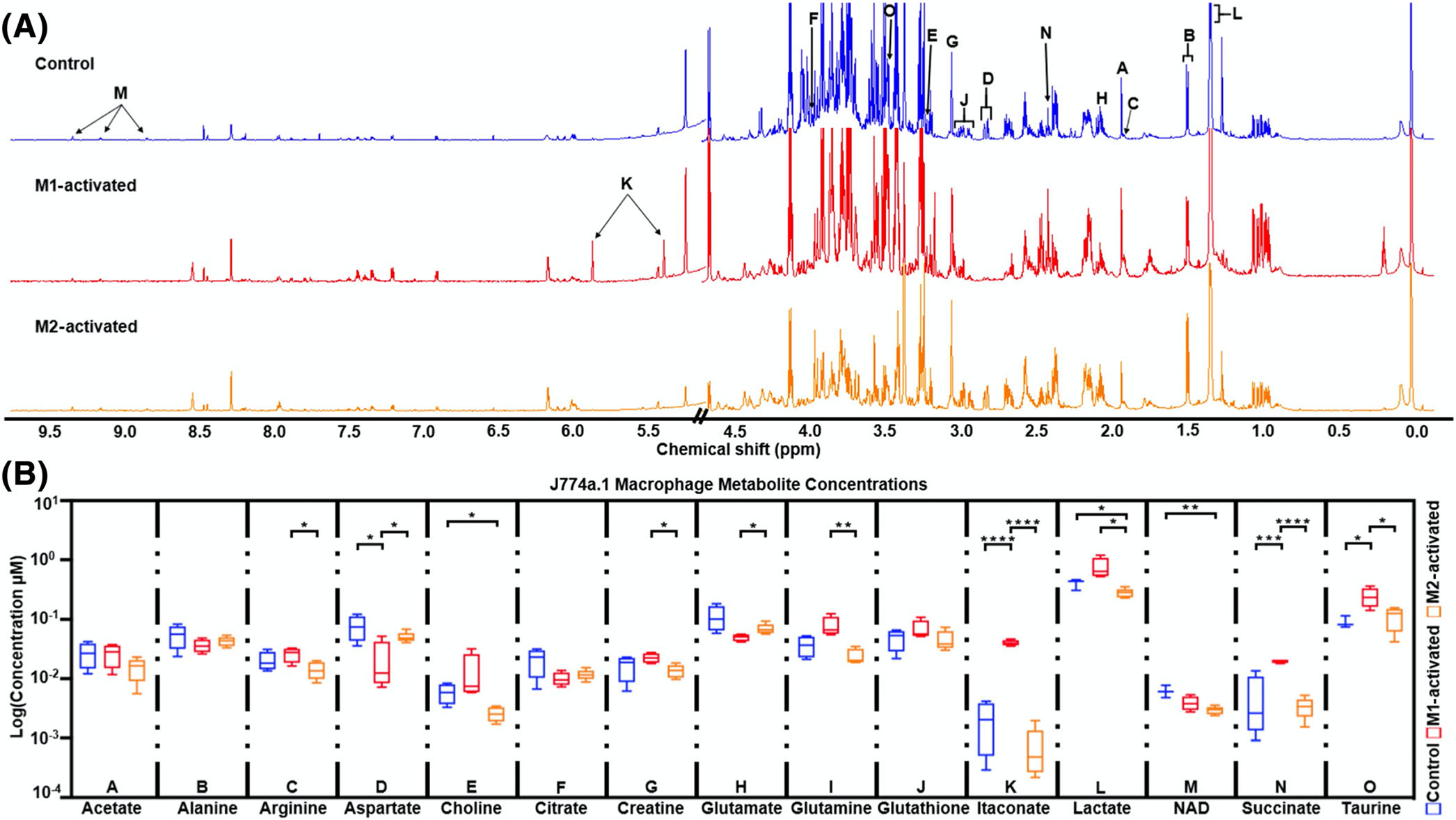
Metabolic differences between M1- and M2-activated macrophages. (A) Representative high-resolution ^1^H MR 1D NOESY spectra of control (blue), M1-activated (red), and M2-activated (orange) J774a.1 macrophage extracts. (B) Metabolite concentrations between control (blue), M1-activated (red), and M2-activated (orange) determined from ^1^H MR data (* *p* < 0.05, ** *p* < 0.01, *** *p* < 0.001, **** *p* < 0.0001). Reproduced in accordance with the Creative Commons CC BY license from^117^

Macrophages are one of the most abundant immune cells in the tumor microenvironment. TAMs play a critical role in tumor progression. Based on their phenotypes and functions, macrophages are thought to be pro-inflammatory or M1, or anti-inflammatory or M2. M1 macrophages play a role in the removal of pathogens and tumor cells, while M2 macrophages are important for clearance of parasites and homeostasis, wound healing, and anti-inflammation.^116^ TAMs tend to be M2-skewed because they possess many characteristics of M2 macrophages.^116^ Hyperpolarized ^13^C MRS of pyruvate and dehydroascorbic acid (DHA) was used to study M1 and M2 macrophage activation.^117^ The study was performed using J7774a.1 macrophages treated with either lipopolysaccharide (LPS) (M1) or interleukin (IL) 13 (M2). Metabolic conversion of pyruvate and DHA was higher in M1 macrophages compared with M2 and nonactivated macrophages (Figure 5). ^1^H MRS identified an increase of itaconate succinate, and lactate in M1 macrophages.^117^ In another study performed on mature monocyte-derived macrophages obtained from healthy donor whole blood, M1 macrophages were obtained by LPS plus IFN-γ stimuli, and M2a macrophages with IL4 stimulus. M1 macrophages were characterized by an increase in oxidative stress and a decrease in mitochondrial respiration. Conversion of glucose to pyruvate and pyruvate to lactate increased in M1 and M2a compared with M0 macrophages.^118^

### 3.3 | Immune cells

Although interest in the metabolism of immune cells has significantly increased with recent advances in immune checkpoint inhibitors and the activation of immune surveillance for cancer treatment, MRS studies of normal and transformed T and B lymphocytes were performed almost 4 decades ago.^119^ Studies with human T lymphocytes purified in culture or entrapped inside agarose beads, and induced by phorbol-12-myristate-13-acetate and ionomycin, were analyzed with ^31^P, ^13^C, and ^1^H NMR spectroscopy.^120^ A decrease of phosphomonoesters and an increase of phosphodiesters was observed after stimulation, together with an increase of glycolysis.

More recently, the conversion of hyperpolarized ^13^C pyruvate to ^13^C lactate was found to significantly increase in activated human T cells compared with resting T cells.^121^ These T cells were isolated from healthy donor blood, and stimulated with anti-TCR/CD3 antibodies, anti-CD28 antibodies, and IL2. The effects of culture media composition on neutrophils were identified using ^1^H MRS with high fatty acid environments resulting in sequestration of palmitic acid into triacylglycerol in the plasma membrane.^122^

### 3.4 | Endothelial cells

Endothelial cell proliferation is necessary during tumor angiogenesis. Studies characterizing the metabolism of endothelial cells are important to understand how depletion of substrates and hypoxia found in tumors may alter endothelial cell metabolism and contribute to the abnormal vasculature found in tumors. In studies performed with human umbilical vein endothelial cells (HUVECs), treating HUVECs with conditioned medium from MDA-MB-231 human breast cancer cells significantly altered the choline phospholipid metabolism of the HUVEC cells, as detected in high-resolution ^1^H MR spectra of these cells.^123^ Understanding the impact of cancer cells on endothelial cell metabolism may provide new insights into how metabolism can be altered to modify tumor vasculature.

## 4 | BIOFLUIDS

### 4.1 | Tumor interstitial fluid

Tumor interstitial fluid (TIF) contains the tumor secretome and forms an essential component of the tumor microenvironment. Altered metabolites in TIF collected from implanted rat tumors were identified by conventional methods using an interstitial fluid collection chamber as previously described.^124^ More recently, the collection chamber was miniaturized to collect TIF from human tumor xenografts implanted in mice.^125^ TIF collected from COX-2 overexpressing triple negative SUM-149 human breast cancer xenografts showed significantly increased levels of lactate, glutamate, acetate, and succinate, and decreased levels of glucose, glutamine, citrate, formate, and lipids.^126^ These results provided new insights into the role of COX-2 and inflammation in the metabolic secretome and tumor metabolism. As the role of metabolism is increasingly being investigated to understand why cancers evade immune surveillance, the relationship between TIF metabolites and anergized T-cells may identify metabolic strategies to improve immune surveillance.

### 4.2 | Body fluids

High-resolution ^1^H MRS has been applied to analyze biofluids such as plasma,^103,127^ urine,^128^ seminal plasma,^129^ and ascites^130^ to evaluate the feasibility of cancer detection, diagnosis, and treatment response monitoring. Biofluids are easily accessible, providing an attractive option for cancer screening, especially for cancers with no available screening method. The potential of ^1^H MRS and ^31^P MRS of serum metabolite markers to detect cancer has been evaluated in different tumor types, including prostate, pancreas, lung, and renal cancers, and leukemia.^103,111,127,131^

Metabolomic profiles in serum from lung cancer patients were found to predict overall survival. Prolonged survival was associated with increased glutamine, valine, and glycine, and reduced glutamate and lipids.^111^ A recent study has highlighted the potential role of HR MAS MRS of serum in the diagnosis and prediction of overall 5-year patient survival in nonsmall cell lung cancer.^132^ Analysis of serum from individuals with acute myeloid leukemia and acute lymphoblastic leukemia identified alterations in metabolic pathways that included glycolysis, TCA cycle, lipoprotein metabolism, and choline and fatty acid metabolism in these two subtypes of acute leukemia.^127^ In pancreatic cancer, lower plasma choline levels were observed in patients with PDAC compared with those in normal subjects and with those in patients with benign pancreatic disease.^103 31^P MRS was applied to plasma samples to assess the systemic phospholipid profile in renal cell carcinoma patients.^131^ Differences in lysophosphatidylcholine concentration were identified between cancer patients and healthy volunteers, as well as an association between phospholipid concentration and tumor stage and metastatic spread.^131^

Distinct urinary metabolic signatures have been observed in esophageal cancer patients, such as changes in fatty acid metabolism, glycolysis, the TCA cycle, and glutaminolysis, with alterations in levels of taurine, glycine, glucose, glutamate, citrate, and creatine that could potentially be used for cancer detection.^128^

Treatment efficacy monitoring can also be followed noninvasively, as shown in recent studies of breast,^133^ head and neck,^134^ and colorectal^135^ cancer, by analyzing either serum or urine samples. Systemic metabolic effects of chemotherapy were analyzed in breast cancer patients’ plasma using ^1^H MRS. The patients were randomized to receive neoadjuvant chemotherapy with or without the antiangiogenic drug bevacizumab.^133^ The study showed that patients who received bevacizumab and chemotherapy showed lower levels of leucine, acetoacetate, and tri-hydroxybutyrate and higher levels of formate compared with patients treated only with chemotherapy. The most important metabolites to discriminate between patients with a good response and patients with a poor response to treatment were citrate, phenylalanine, and histidine.^133^ The potential of using urine samples to predict adverse effects and response to chemotherapy was evaluated in colorectal cancer patients.^135^ While the study showed promising results, it was limited by a small sample size, and further studies are needed to confirm the predictors identified.

## 5 | CANCER-INDUCED CHANGES IN ORGAN METABOLISM

**FIGURE 6:**
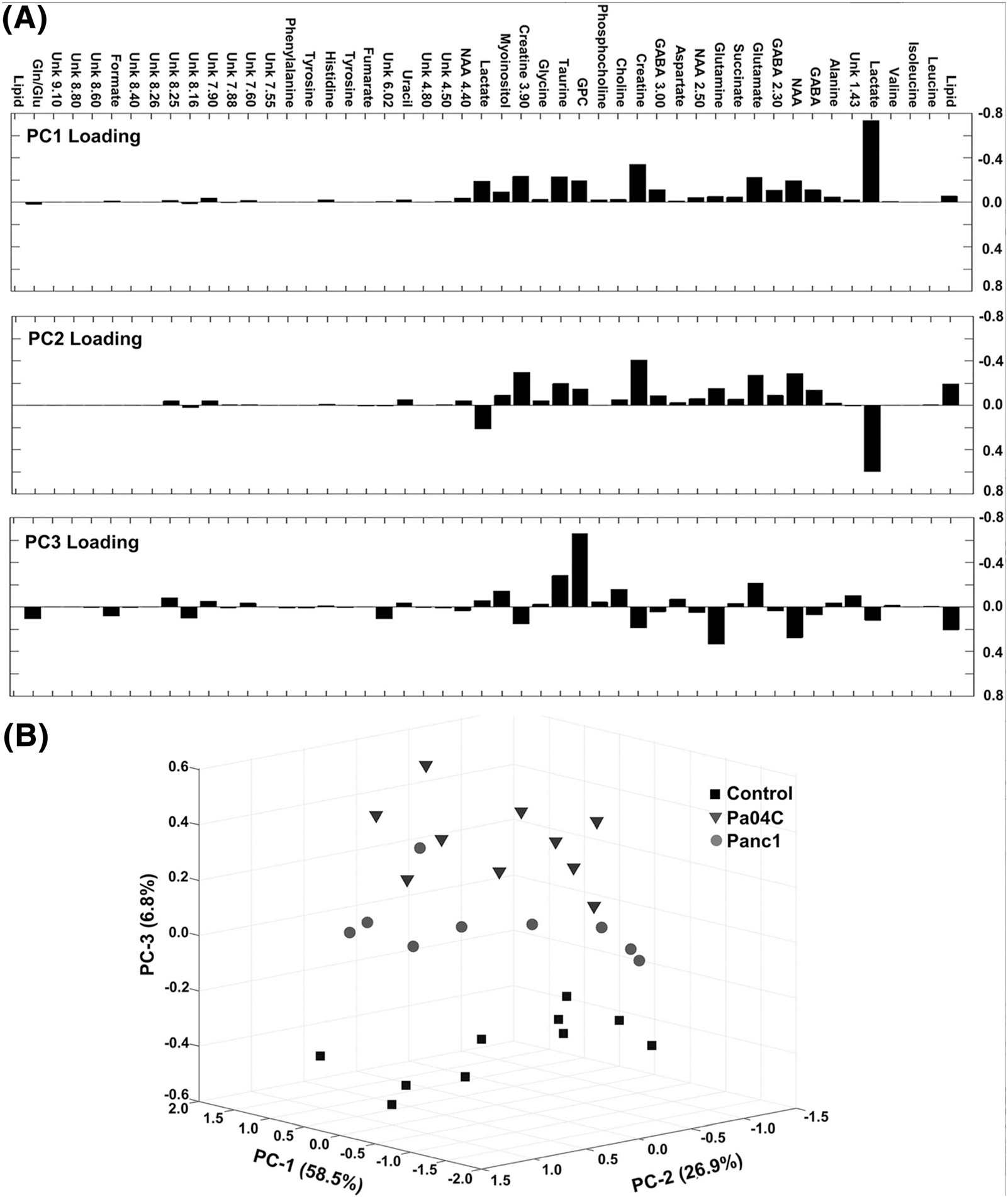
Brain metabolites from nontumor-bearing control mice, and Panc1 pancreatic tumor-bearing and Pa04C cachectic pancreatic tumor-bearing mice, analyzed by ^1^H MRS. (A) Principal component loadings generated from principal component analysis (PCA) of the overall metabolic profiles acquired from aqueous-phase mouse brain ^1^H MR spectra. (B) Three-dimensional PCA representation of the quantitative data-derived score plots of aqueous phase mouse brain extracts, showing differential clustering of each group: control mice (squares), Pa04C tumor-bearing mice (triangles), and Panc1 tumor-bearing mice (circles). GABA, γ-aminobutyric acid; Gln/Glu, glutamine/glutamate; NAA, N-acetyl aspartate; Unk, unknown. Reproduced in accordance with the Creative Commons CC BY license from^103^

A largely overlooked but critically important aspect of cancer is its impact on the body and its organs. Cancer cells have mechanisms such as secretion of cytokines and exosomes to send systemic signals.^136,137^ An example of the systemic changes induced by cancers is cancer-induced cachexia, a syndrome that results in unexplained weight loss, morbidity, and mortality.^138^ Ex vivo high-resolution MRS can be applied to understand how cancers alter the metabolism of critical organs. In a recent study,^103^ significant changes in brain metabolites in the cholinergic and glutamatergic pathways were caused by a cachexia-inducing human PDAC xenograft that may explain the morbidity associated with cachexia. A noncachexia-inducing xenograft also induced changes in brain metabolism, highlighting the impact of cancers on organ metabolism. PCA analysis of brain ^1^H MRS spectra obtained from normal mouse brains, and brains from cachexia (Pa04C) and noncachexia-inducing (Panc1) human PDAC xenografts, showed distinct differential clusters based on distinct metabolic signatures (Figure 6). Ongoing studies are expanding these investigations to other organs including the spleen in immune-competent mice. As changes in organ metabolism are identified, human studies with noninvasive spectroscopic imaging can be applied to understand how cancers alter organ metabolism.

Ex vivo MRS and HR MAS MRS provide multiple advantages such as detection of multiple metabolites, and the ability to compare metabolic information with molecular and histological analyses. Some of the limitations include limited sensitivity of detection compared with techniques such as mass spectrometry, supporting the combination of MRS with more sensitive detection techniques for applications. Combining metabolic profiles derived from liquid chromatography–mass spectrometry with MRS allowed the distinguishing of serum from esophageal adenocarcinoma patients compared with patients with Barret’s esophagus, high-grade dysplasia, and healthy individuals.^139^ The invasiveness of acquiring biopsies and the limited tissue sampling of biopsies, especially in the context of tumor heterogeneity, are other limitations. However, good agreement was observed in an astrocytoma study comparing ex vivo tumor biopsy HR MAS ^1^H MR spectra with the in vivo tumor metabolic profile when viable tumor regions were compared.^140^

## 6 | FUTURE DIRECTIONS

### 6.1 | Understanding immunometabolism

The achievement of lasting control of some cancers with immunotherapy is providing exciting new opportunities for cancer treatment. Efforts to understand why some cancers respond to immunomodulation and others do not are critically important to improve the outcome of immunotherapy. One important area for the future is to apply MRS to understand the metabolism of immune cells, and changes in immune cell metabolism following coculture of immune cells with cancer cells, or following incubation with conditioned medium obtained from cancer cells. Such insights may lead to metabolic modulation strategies to create a more active immune surveillance of cancer cells.

### 6.2 | Glutamine metabolism

Many cancer cells, including pancreatic cancer cells, are glutamine addicted. Targeting glutamine metabolism presents an exciting opportunity for treatment. Multinuclear MRS of cell and tissue extracts of cells with specific enzymes downregulated or overexpressed in the glutamine metabolism pathway can provide an understanding of metabolic networks altered by changes in glutamine metabolism, and their impact on cancer cell survival. Such insights can lead to the development of novel metabolic treatment strategies.

### 6.3 | Applications of AI to ex vivo MRS

Advances in computational capabilities and novel algorithms to augment data and analyze large datasets create new possibilities for the applications of AI to ex vivo MRS in cancer detection and treatment. Metabolite-rich datasets of the entire spectral range obtained with high-resolution MRS provide the ideal data for artificial neural network analyses. The ease of obtaining biofluids such as plasma/serum or urine make the integration of AI with MRS data from biofluids that contain spectral information of metabolites and proteins/lipoproteins an attractive area in screening for cancers, as well in predicting and monitoring response to therapy. Such tests can be combined with traditional noninvasive imaging and with other blood-based tests to increase specificity and sensitivity.

### 6.4 | Focus on liquid cancers

While solid tumors have been extensively investigated, the applications of ex vivo MRS to liquid cancers have not been as extensive. An expanded focus on liquid cancers can provide new insights into the role of metabolism in malignant transformation.

### 6.5 | Understanding cancer-associated stromal cells such as CAFs and macrophages under well-controlled physiological conditions

Stromal cells such as fibroblasts and macrophages play a role in cancer progression, drug resistance, and immunosuppression. Multinuclear ex vivo MRS of stromal cells can provide insights that can be incorporated into strategies to target stromal cells found in cancers to make cancers more vulnerable to treatments.

### 6.6 | Metabolotheranostics

Ex vivo MRS can provide a metabolic fingerprint of cancers so that cancer-based alterations in metabolism can be specifically targeted with image-guided platforms delivering siRNA or cDNA that can be used to design personalized treatment. Research into metabolic changes induced by cancers in vital organs in immune-competent mice will expand research into modifying organ metabolism to improve cancer treatment outcomes.

## Data Availability

Data sharing not applicable to this article as no datasets were generated or analysed during the current study.

## Abbreviations used:

2-HG: 2-hydroxyglutarate
6-PG: 6-phosphogluconate
AI: artificial intelligence
ASCT2: alanine, serine, cysteine transporter 2
CAFs: cancer-associated fibroblasts
CEST: chemical exchange saturation transfer
Chk: choline kinase
COX-2: cyclooxygenase-2
CV: crossvalidation
DHA: dehydroascorbic acid
ECM: extracellular matrix
Etnk: ethanolamine kinase
G6PDH: glucose-6-phosphate dehydrogenase
GLS: glutaminase
GPC: glycerophosphocholine
HR MAS: high-resolution magic angle spinning
HUVECs: human umbilical vein endothelial cells
IDH: isocitrate dehydrogenase
IL: interleukin
LGOGs: low-grade oligodendrogliomas
LPS: lipopolysaccharide
MRI: magnetic resonance imaging
MRS: magnetic resonance spectroscopy
NHA_ALT_: NHAs that lack TERT and use the ALT pathway
NHAs: normal human astrocytes
PC: phosphocholine
PCA: principal component analysis
PCAFs: prostate cancer-associated fibroblasts
PDAC: pancreatic ductal adenocarcinoma
PDC: pyruvate dehydrogenase complex
PD-L1: program death ligand 1
PDX: patient-derived xenograft
PE: phosphoethanolamine
PGK1: phosphoglycerate kinase 1
pHe: extracellular pH
PLS-DA: partial least discrimination analysis
PPP: pentose phosphate pathway
siRNA: small interfering RNA
TAMs: tumor-associated macrophages
TCA: tricarboxylic acid
tCho: total choline
TERT: telomerase reverse transcriptase
TIF: tumor interstitial fluid
